# Impact of the Family Health Program on the quality of vital information and reduction of child unattended deaths in Brazil: an ecological longitudinal study

**DOI:** 10.1186/1471-2458-10-380

**Published:** 2010-06-29

**Authors:** Davide Rasella, Rosana Aquino, Mauricio L Barreto

**Affiliations:** 1 Instituto de Saúde Coletiva, Federal University of Bahia, Rua Basílio da Gama s/n - Canela, 40. 110-040 Salvador, Bahia, Brazil

## Abstract

**Background:**

Vital information, despite of being an important public health instrument for planning and evaluation, in most of the developing countries have still low quality and coverage. Brazil has recently implemented the Family Health Program (PSF), one of the largest comprehensive primary health care programs in the world, which demonstrated effectiveness on the reduction of infant mortality. In the present study we evaluate the impact of the PSF on mortality rates related to the quality of vital information: the under-five mortality rate due to ill-defined causes and unattended death.

**Methods:**

Data on mortality rates and PSF coverage was obtained for the total 5,507 Brazilian municipalities from 2000 to 2006. A multivariate regression analysis of panel data was carried out with a negative binomial response by using fixed effects models that control for relevant covariates.

**Results:**

A statistically significant negative association was observed between PSF coverage levels, classified in none (0%, the reference category), low (<30.0%), intermediate (≥ 30.0% and <70.0%) and high (≥ 70.0%), and all analysed mortalities rates, with a reduction of 17% (Rate Ratio [RR]: 0.83; 95% confidence interval [CI]: 0.79 - 0.88), 35% (RR: 0.65; 95% CI: 0.61-0.68) and 50% (RR: 0.50; 95% CI: 0.47-0.53) on under-five mortality due to ill-defined causes, respectively. In the mortality rate for unattended death the reduction was even greater, reaching 60% (RR: 0.40; 95% CI: 0.37-0.44) in the municipalities with the highest PSF coverage. The PSF effect on unattended deaths was slightly stronger in municipalities with a higher human development index.

**Conclusions:**

The PSF, a primary health care program developed mostly in rural and deprived areas, had an important role on reducing the unattended deaths and improving the quality of vital information in Brazil.

## Background

Vital statistics are essential instruments for planning and evaluation of public health policies. Despite the fact that in the past sixty years many efforts have been made by national and international institutions to improve the quality and availability of vital statistics, they are still, especially in the developing countries, in a deplorable state[1]. A cause of death is assigned for no more than one third of the total deaths worldwide, and there is often considerable uncertainty about the diagnosis. The inequalities in registration of vital events are large: developing countries account for 99% of all the unregistered births, concentrated mainly in southern Asia and sub-Saharan Africa[2]. Epidemiological estimates, obtained by extrapolation from household surveys, are currently used in various countries to understand the probable values of key health indicators, including vital information[3]. Despite their utility, they should not be considered as substitutes of reliable civil registration data.

Various methods have been developed for the assessment of a country health information system, and, in terms of the quality of death information, one of the most used criteria is the proportion of deaths classified into ill-defined categories [4].

In Brazil, the Ministry of Health created in 1976 the Mortality Information System (SIM - acronym in Portuguese), based on the official death certificates[5]. Over the last two decades the coverage and the quality of the mortality data has improved substantially, but due to the incompleteness of the vital registration systems in the less developed regions, it is still not possible to estimate the mortality rates directly. However recent analyses indicate that almost 80% of the Brazilian population lives in areas with satisfactory levels of death information[6].

In 1994 the Minister of Health adopted the Family Health Program (PSF - acronym in Portuguese) as strategy to convert the model of care toward the primary health care and increase the National Health System coverage, especially for deprived areas (such as rural communities and urban slums)[7]. In this period the PSF has experienced a dramatic expansion, and by the end of 2008 it was present in 94% of the Brazilian municipalities, with coverage of almost 50% of the total national population[8]. The PSF organization is strongly decentralized and is managed, following national regulations, at the municipality level. The program operates through multiprofessional teams (ESF - acronym in Portuguese). Each ESF, which is at the minimum composed by a physician, a graduated nurse, an auxiliary nurse and 4 to 6 community health workers (CHW), attends to an established geographic area of about 3,450 people [7].

The PSF created in 1998 its own information system (SIAB - acronym in Portuguese), which collects data about hygiene, housing conditions, health situation and vital information of the families under the responsibility of the ESF[9].

Despite the fact that the PSF, and consequently the SIAB, has a wide national coverage, few local studies have analyzed the improvement of the vital statistics in municipalities which adopted this program[10,11].

The aim of the present study is to evaluate the impact of the PSF on the under-five mortality due to ill-defined causes, in the Brazilian municipalities from the year 2000 to 2006, decomposing its effect on unattended deaths and remaining ill-defined categories.

## Methods

This is an ecological study, adopting the municipalities (counties) as unit of analysis. We used a panel data or longitudinal data model, creating a panel dataset from several databases for the years 2000 until 2006.

We used the data of the total 5,507 Brazilian municipalities during the 7-year period.

All the mortality rates (ill-defined causes of death, unattended death and remaining ill-defined causes of death) were obtained by direct calculation, using the Brazilian death certificates database for each year of the study, selecting the deaths per age and/or cause and combine them per municipality. For selected causes we used the following WHO International Classification of Disease (ICD-10) categories [12]: Ill-defined causes of death (chapter XVIII, R00-R99), unattended death (R98) and remaining ill-defined categories (ill-defined causes of death without unattended deaths, R00-R97, R99). The yearly coverage of the PSF was calculated as the ratio of the population covered by the program to the total population of the same municipality, and it was classified in 4 different categories: without PSF, low coverage (less than 30.0% of population), intermediate coverage (from 30.0% to 69.9%), and high coverage (greater than or equal to 70.0%).

We selected a set of variables (available in the source datasets) identified as determinants of the quality of vital information and as socioeconomic characteristics of the municipalities, using them as covariates for the different models: total fertility rate (stratified as ≤ 2.42 and >2.42 children per childbearing-age woman, median of the distribution), per capita income (in Brazilian real [BR$], stratified as ≤ BR$ 120 and >BR$ 120, the national poverty threshold), population (stratified as ≤ 50,000 and >50,000 inhabitants, a common cutoff value for municipality dimension) and percentage of illiterates among individuals older than 15 years (stratified as ≤ 15.4% and >15.4%, median of the distribution).

### Data Sources

The data used in this study were collected from different information systems. We obtained the vital statistics, made available by the Brazilian Ministry of Health, from the Mortality Information System (SIM) and Information System on Lives Births (SINASC)[13]. For the population covered by the PSF in each municipality we used the Primary Care Information System (SIAB). For the socioeconomic and demographic variables we used the data from the Brazilian Institute of Geography and Statistics (IBGE)[14] and from the Human Development Atlas of the United Nation Development Program[15]. Since these covariates were obtained from the 1991 and 2000 national censuses databases, the annual values from 2001 to 2006 were calculated by linear extrapolation,[16] with the exception of the population (IBGE projection).

### Statistical analysis

For this study we used a multivariable negative binomial regression for panel data with fixed effects specification. A model for each different mortality rate category, which represents the dependent variable, was fitted using the PSF coverage as main independent variable and a set of demographic, social and economic determinants as covariates.

Negative binomial regression is used when the outcome to be analyzed is counting and the Poisson model assumption that the mean is equal to variance is not met, usually because the data present a greater dispersion[17]. Panel data models introduce two different terms to control first for unobserved time-invariant characteristics of each panel, and second for the time-varying characteristics of the panels according to the year. The choice between the fixed and random effects models is based on the method in which the sample is drawn and on a specification test, the Hausman tests[18]. According to these criteria, the more appropriate specification for all the models was the fixed effect.

To evaluate the association between specific mortality rates and the PSF coverage levels, we calculated the mortality rate ratios, both crude and adjusted for the covariates, using the municipalities without PSF as reference category.

The number of observations varied in different models due to the presence of municipalities with zero specific deaths count for the all 7-year period. For statistical reasons, linked to the computation of the fixed effect models, [19] these municipalities were not included in the model fitting.

The municipalities were successively stratified by the human development index (HDI), which is composed of measures of life expectancy, education and standard of living (≤ 0.713 and >0.713; 0.713 was the Brazilian median for the year 2000).

No ethics committee approval was needed for our study, being the data used in this research obtained from a public-use data set.

For the statistical analysis and database processing we used the software Stata version 10.1.

## Results

In the period from 2000 to 2006, in Brazil, the under-five mortality rate decreased from 24.7 to 19.3 per 1000 live births (Table 1). The mortality rate due to ill-defined causes, which represented the 12.9% of the total under-five mortality in 2000, decreased considerably (from 3.2 per 1000 live births in 2000 to 1.0 in 2006). The mortality rate for unattended death, which constituted the 8.4% of the total under-five deaths and the 64.9% of the deaths for ill-defined causes in 2000, reduced considerably, decreasing from 2.1 to 0.4 per 1000 live births.

**Table 1:** Mortality rates per 1000 live births (MR) and under-five death proportion (%) for causes groups: Brazil, 2000-2006.

	2000		2001		2002		2003		2004		2005		2006	
	**MR**	**%**	**MR**	**%**	**MR**	**%**	**MR**	**%**	**MR**	**%**	**MR**	**%**	**MR**	**%**

Under-five mortality	24.7	100.0	23.3	100.0	22.5	100.0	22.2	100.0	20.9	100.0	19.8	100.0	19.3	100.0
Ill-defined cause mortality*	3.2	12.9	2.6	11.0	2.2	9.9	2.1	9.4	1.7	7.9	1.2	6.2	1.0	5.2
Unattended deaths mortality*	2.1	8.4	1.5	6.6	1.4	6.1	1.2	5.3	0.9	4.3	0.6	2.9	0.4	2.0

* under-five mortality

PSF coverage rapidly increased during the period (Table 2): the number of municipality without PSF decreased from 2,843 in 2000 to 367 in 2006, and these with coverage more than or equal to 70% increased from 851 to 3,432.

**Table 2:** Independent variables medians for the analyzed municipalities (5507): Brazil, 2000-2006.

Variable	2000	2001	2002	2003	2004	2005	2006
PSF coverage	0.0	30.4	52.6	64.1	70.9	80.0	85.3
Total fertility rate	2.67	2.59	2.49	2.42	2.34	2.26	2.19
Percentage of illiterates among individuals older than 15 years	18.0	17.1	16.1	15.2	14.3	13.5	12.5
Per capita income (Brazilian Reais)	159.1	164.1	168.7	174.0	178.8	183.6	188.3
Population	10418	10446	10513	10577	10601	10744	10806

Socioeconomic conditions improved nationally: there were reductions in the total fertility rate and in the percentage of illiterate persons aged 15 years or more, while increases were observed in the monthly per capita income.

Table 3 shows the effect of the PSF coverage levels on the mortality rate ratios, crude and adjusted for the covariates. In the adjusted model for the ill-defined mortality rate ratio, reductions of 17%, 35% and 50% (all statistically significant) were found for the low, intermediate and high level of coverage, respectively.

**Table 3:** Fixed-Effect Negative Binomial Models for the crude and adjusted association between specific under-five mortality groups and PSF coverage: Brazil, 2000-2006.

Variables	Ill-defined Mortality RR (95% CI)				Unattended death Mortality RR (95% CI)		Remaining Ill-defined Mortality RR (95% CI)	
	Crude		Adjusted					
*PSF coverage*								
No PSF ^a^	1		1		1		1	
Low ^b^	0.78	(0.74-0.82)	0.83	(0.79-0.88)	0.74	(0.69-0.80)	0.98	(0.91-1.05)
Intermediate ^c^	0.56	(0.53-0.58)	0.65	(0.61-0.68)	0.57	(0.53-0.62)	0.80	(0.74-0.87)
High ^d^	0.42	(0.40-0.45)	0.50	(0.47-0.53)	0.40	(0.37-0.44)	0.74	(0.68-0.81)
Total fertility rate >2.42 children per childbearing-age woman			1.56	(1.49-1.64)	1.73	(1.61-1.85)	1.36	(1.26-1.46)
Percentage of illiterates among individuals older than 15 years >15.4%			1.06	(0.98-1.14)	1.31	(1.17-1.47)	1.02	(0.92-1.13)
Per capita income >BR$ 120			0.81	(0.75-0.88)	0.71	(0.64-0.79)	0.91	(0.80-1.04)
Population >50,000 inhabitants			0.35	(0.30-0.39)	0.29	(0.25-0.34)	0.36	(0.30-0.44)
Num. Observations		28168		28168		19278		23261
Num. Municipalities		4024		4024		2754		3323

Abbreviations: CI = Confidence Interval; PSF = Family Health Program; RR = Rate Ratio.

^a ^= defined as coverage equal to 0% of the population.

^b ^= defined as coverage of less than 30% of the municipal population.

^c ^= defined as coverage of 30% to 69.9% of the municipal population.

^d ^= defined as coverage of more than or equal to 70% of the municipal population.

The PSF effect was greater in the mortality for unattended death, percentage reductions were 26%, 43% and 60%, respectively, for increasing levels of coverage. We observed the smallest effect in the mortality for the remaining ill-defined causes, reaching a maximum reduction of 26%.

Table 4 shows the effect of the PSF coverage levels on the mortality rate ratios for the municipalities stratified by the human development index. The less developed municipalities (with the lower HDI) had a higher percentage of under-five unattended deaths and deaths for ill-defined causes in the period of 2000-2006 (12.7% and 17.3% respectively) in comparison with the more developed municipalities (1.3% and 4.8%). The reduction in the mortality rate for unattended death was slightly higher in the municipalities with better HDI, reaching a maximum of 64% for the high level of coverage. The PSF effect on the mortality for the remaining ill-defined causes of death was similar in the two subsets of municipalities.

**Table 4:** Fixed-Effect Negative Binomial Models for the adjusted* association between specific under-five mortality groups and PSF coverage, by Human Development Index (HDI): Brazil, 2000-2006.

Variables	Unattended death Mortality RR (95% CI)		Remaining Ill-defined Mortality RR (95% CI)	
*HDI *≤ *0.713*				
PSF coverage				
No PSF ^a^	1		1	
Low ^b^	0.75	(0.69 - 0.81)	1.02	(0.91 - 1.14)
Intermediate ^c^	0.59	(0.55 - 0.64)	0.85	(0.75 - 0.95)
High ^d^	0.42	(0.38 - 0.45)	0.76	(0.67 - 0.86)
Num. Observations	14154		12936	
Num. Municipalities	2022		1848	
				
*HDI >0.713*				
PSF coverage				
No PSF ^a^	1		1	
Low ^b^	0.69	(0.59 - 0.81)	0.95	(0.86 - 1.05)
Intermediate ^c^	0.49	(0.41 - 0.59)	0.79	(0.71 - 0.88)
High ^d^	0.36	(0.29 - 0.46)	0.80	(0.69 - 0.92)
Num. Observations	5124		10325	
Num. Municipalities	732		1475	

Abbreviations: CI = Confidence Interval; PSF = Family Health Program; RR = Rate Ratio.

^a ^= defined as coverage equal to 0% of the population.

^b ^= defined as coverage of less than 30% of the municipal population.

^c ^= defined as coverage of 30% to 69.9% of the municipal population.

^d ^= defined as coverage of more than or equal to 70% of the municipal population.

*Models adjusted for total fertility rate, per capita income, percentage of functional illiterates among individuals older than 15 years and population of the municipalities.

## Discussion

Our study demonstrates that the PSF succeeds in improving the quality of information on the causes of death and to reduce the number of unattended deaths in the Brazilian municipalities in the years 2000 to 2006.

Brazil has been considered, according to a recent international assessment, among the countries with a medium level of quality on death information [20]. This was due to the insufficient level of completeness and to the relatively high proportion of deaths due to ill-defined causes, in particular in the North and Northeast regions of the country[21]. Indeed, in municipalities of these regions, situations of extreme poverty are quite common, and many children die at home without any medical assistance, being buried in the household backyards or in unofficial cemeteries[22].

The main current flaws of the SIM on child death registration are: occurrence of deaths without completion of the death certificate, absence of strategies for identification and certification of household deaths, incorrect classification of fetal and neonatal deaths, completion of the certificates by non-doctors and problems with the codification and flow of the mortality data[6].

Our results confirm suggestions from previous local studies that the implementation of the PSF in a municipality could improve the coverage and quality of the vital information [6,10,11,22]. In many rural municipalities the SIAB has a higher coverage than the SIM, because the CHW during its routine activities succeeds in collecting vital information in the community more efficiently that the traditional mortality information system[23]. The community health workers, as other health professionals, can compile a birth certificate (contributing in this way to improve the coverage of the SINASC) [24], but they are not allowed to complete a death certificate, which is the physician's prerogative[25]. However, the CHW could encourage the community to declare births or deaths to public notary offices and in some cases they can complete their own death notifications, without detailing the cause of death, in order to improve the coverage of the mortality data[23]. In various Brazilian states projects of integration of the SIAB, SIM and SINASC have been implemented with positive results, especially in the Northeast region[26].

The strong effect of the PSF on the reduction of the unattended deaths is of particular relevance, considering that the unattended deaths constitute the major part of the mortality for ill-defined causes and that they had a dramatic reduction between the year 2000 and 2006.

An unattended death is a "death in circumstances where the body of the deceased was found and no cause could be discovered",[12] this cause is usually unknown because of lack of medical care at death or during the illness or condition leading to death[27]. This kind of death notification implies a great distance between the community and the heath system, that can be geographical or even socio-cultural. Especially in less developed countries, the proportion of unattended deaths (and of the others ill-defined categories) can be considered as an indicator of the access to medical care,[27,28] and should be one of the first to be reduced by effective primary health care programs.

Indeed, the active home visits of the community health workers, and in some cases of the physicians, [29] could determine the serious health conditions of a child, and guarantee immediate medical assistance or a transfer to hospital. This implies not only an improvement on the quality of cause of death notification (in the case of subsequent child death), but hopefully the possibility to avoid this death, considering that the majority of those domiciliary deaths are for easy preventable causes, as diarrhoeal diseases[10].

As a matter of fact, recent studies demonstrate that the PSF succeeds in reducing the infant and child mortality, especially for vulnerable causes (like diarrhea) [30,31].

The difference of the PSF effect on reducing the unattended deaths, in comparison with the remaining ill-defined causes of death, could be explained by the fact that the last ones are more difficult to reduce, depending more on the current medical knowledge, diagnostic errors, deficiencies in certification, coding and information processing problems[27]. However, the proximity of the PSF team, in particular of the CHW, with the community, and the fact that the SIAB records the health history of each individual of its respective area, could facilitate a proper identification of the basic cause of death[32].

The slightly lower effect of the PSF on reducing the unattended deaths in the less developed municipalities, which host the greater part of the Brazilian unattended deaths, could be explained by the fact that these areas are mostly rural, with difficult geographical access and socio-cultural barriers, diminishing the effectiveness of the PSF actions. Additionally the community health agents could promote the declaration of the numerous hidden domiciliary deaths, therefore increasing the number of unattended deaths.

The lower impact of the PSF on specific causes of mortality in less developed municipalities does not necessarily contradict the demonstrated PSF effect on reduction of health inequalities [31]. As a matter of fact poor counties have usually highter proportional mortality for preventable causes, as diarreia or unattended death, on which PSF has a stronger effect, [32] and even if the effect on these specific causes is lower than in the richer areas, the resulting global impact on infant mortality is greater.

The similar PSF effect on the mortality due to the remaining ill-defined categories in the two subsets of municipalities confirms the differences between these categories and the unattended deaths.

The main limitation of this study is the use of all the Brazilian municipalities without any selection for the coverage of the vital information. As a matter of fact the validity of the distribution by causes is affected by the under-registration of deaths. There is a complex relationship between the increase in the coverage of death notification, as occurred in Brazil in the last years, and the possible correspondent variation of the vital information quality, determining in some cases an increase in the proportion of ill-defined causes, and in others a decrease[27,33]. To demonstrate the robustness of our results, we performed an additional analysis in a subset of municipalities selected for the adequacy of vital information (both in terms of coverage and quality of vital information), obtaining similar estimates of the PSF effect in the reduction of unattended deaths and deaths for ill-defined causes.

As explained in the methods section, in the models the number of observations varies according to the mortality rate for a fixed effect computation reason.

Comparing the municipality excluded with the included, we found slightly different socioeconomic characteristics. We performed a sensitivity analysis of the same models using random effects which calculate the parameters considering all observations, and we obtained estimates similar to the fixed effects models.

To verify that the high variability of the mortality rates during the years in the small municipalities could not affect the validity of our results, we fitted the same models in the subset of municipalities with less than 50,000 inhabitants, obtaining very similar estimates.

The use of extrapolation techniques to estimate the annual value of some covariates could represent another limitation of the study. As a matter of fact the only measured values of these variables were from the census for 1991 and 2000, and the values from 2001 to 2006 have been obtained by extrapolation. Possible bias introduced by the use of the crude extrapolation instead of more complex estimations techniques are limited by the categorization of the variables that could smooth sharp fluctuations artificially introduced by the method. Moreover, comparisons of the values of the covariates, obtained by extrapolation at the state level, with the values of the National Household Sample Survey (PNAD), which provide yearly estimates of these variables for each Brazilian state, [34] demonstrated great similarities.

A time-representing variable was not included in the model because the use of mortality rate ratios with a comparison group (municipalities without PSF) allowed for control of unknown effects linked to mortality time trends[31].

## Conclusions

This study demonstrates the effectiveness of the PSF on the improvement of the vital information quality and on the reduction of unattended deaths. These findings could be explained by the PSF capability to obtain factual information about the health situation of the community and by its prerogative of increasing the population access to medical care. The impressive PSF effect on reducing the number of unattended deaths demonstrates the effectiveness of its approach on closing the gap between the less-developed, rural communities and the national health system. Moreover the PSF ability to reduce the proportion of remaining ill-defined causes of death point out the need for an effective integration of the SIAB, SIM and SINASC information systems.

Nowadays the improvement of the quality and coverage of the vital information systems is a worldwide concern. There is a growing demand for evidence in terms of impact of health and social policies, and these evidences need to be built on reliable birth and death data[35].

One of the most important global health and development initiatives in recent years, the Millennium Development Goals, grounds six of its eight targets on reliable vital statistics[36]. The maintenance and improvement of the infrastructure for vital information systems involve different partners of various sectors, and require supportive advocacy, public trust, legal frameworks and adequate human and financial resources[37]. As suggested in this article, primary health care programs could be integrated with vital information systems and contribute to their progress.

## Competing interests

The authors declare that they have no competing interests.

## Authors' contributions

DR, RA and MLB were involved in the study design. DR and RA were involved in data collection and statistical analysis. All investigators contributed to the interpretation of results and to writing the report. All investigators had access to all data in the study and hold final responsibility for the decision to submit it for publication.

## Pre-publication history

The pre-publication history for this paper can be accessed here:

http://www.biomedcentral.com/1471-2458/10/380/prepub
